# Vancomycin modifies the expression of the *agr* system in multidrug-resistant *Staphylococcus aureus* clinical isolates

**DOI:** 10.3389/fmicb.2015.00369

**Published:** 2015-05-05

**Authors:** Vicenta Cázares-Domínguez, Sara A. Ochoa, Ariadnna Cruz-Córdova, Gerardo E. Rodea, Gerardo Escalona, Alma L. Olivares, José de Jesús Olivares-Trejo, Norma Velázquez-Guadarrama, Juan Xicohtencatl-Cortes

**Affiliations:** ^1^Laboratorio de Investigación en Bacteriología Intestinal, Hospital Infantil de México Federico GómezMéxico DF, Mexico; ^2^Laboratorio de Infectología, Hospital Infantil de México Federico GómezMéxico DF, Mexico; ^3^Posgrado en Ciencias Genómicas, Universidad Autónoma de la Ciudad de MéxicoMéxico DF, Mexico

**Keywords:** *Staphylococcus aureus*, multidrug-resistant, vancomycin, *agr* system, antibiotics

## Abstract

*Staphylococcus aureus* is an opportunistic pathogen that colonizes human hosts and causes a wide variety of diseases. Two interacting regulatory systems called *agr* (accessory gene regulator) and *sar* (staphylococcal accessory regulator) are involved in the regulation of virulence factors. The aim of this study was to evaluate the effect of vancomycin on *hld* and *spa* gene expression during the exponential and post-exponential growth phases in multidrug-resistant (MDR) *S. aureus*.

**Methods:** Antibiotic susceptibility was evaluated by the standard microdilution method. The phylogenetic profile was obtained by pulsed-field gel electrophoresis (PFGE). Polymorphisms of *agr* and SCC*mec* (staphylococcal cassette chromosome *mec*) were analyzed by multiplex polymerase chain reaction (PCR). The expression levels of *hld* and *spa* were analyzed by reverse transcription-PCR. An enzyme-linked immunosorbent assay (ELISA) was performed to detect protein A, and biofilm formation was analyzed via crystal violet staining.

**Results:** In total, 60.60% (20/33) of *S. aureus* clinical isolates were MDR. Half (10/20) of the MDR *S. aureus* isolates were distributed in subcluster 10, with >90% similarity among them. In the isolates of this subcluster, a high prevalence (100%) for the *agr*II and the cassette SCC*mec* II polymorphisms was found. Our data showed significant increases in *hld* expression during the post-exponential phase in the presence and absence of vancomycin. Significant increases in *spa* expression, protein A production and biofilm formation were observed during the post-exponential phase when the MDR *S. aureus* isolates were challenged with vancomycin.

**Conclusion:** The polymorphism *agr*II, which is associated with nosocomial isolates, was the most prevalent polymorphism in MDR *S. aureus*. Additionally, under our study conditions, vancomycin modified *hld* and *spa* expression in these clinical isolates. Therefore, vancomycin may regulate alternative systems that jointly participate in the regulation of these virulence factors.

## Introduction

*Staphylococcus aureus* is an opportunistic pathogen capable of causing a wide variety of diseases in humans, ranging from localized infections of the skin and soft tissues to life-threatening systemic infections (Archer, 1998; Shopsin and Kreiswirth, 2001; David and Daum, 2010; Sowash and Uhlemann, 2014). An infection is initiated when *S. aureus* has access to subcutaneous tissues and is disseminated by the circulatory system, and it infects nearly every organ, leading to severe osteomyelitis, sepsis, abscesses, endocarditis, pneumonia, and toxic shock syndrome (Archer, 1998; Shopsin and Kreiswirth, 2001; David and Daum, 2010; Sowash and Uhlemann, 2014). *S. aureus* can produce a remarkable array of wall surface and secreted virulence factors that contribute to the establishment and maintenance of infection (Novick et al., 1993). These cell surface virulence factors include microbial surface components that recognize extracellular matrix proteins, such as fibrinogen, laminin, plasminogen, vitronectin, fibronectin, thrombospondin, and bone sialoprotein (Falord et al., 2011; Yamamoto et al., 2013). The secreted virulence factors are generally produced during the post-exponential or stationary phase, and they include several extracellular toxins (i.e., alpha-, beta-, gamma-, and delta-hemolysin, enterotoxins, exfoliative toxins A and B, and toxic shock syndrome toxin-1) and exoenzymes (i.e., lipase, nucleases, proteases, hyaluronate lyase, and metalloproteases; Chan and Foster, 1998; Kolar et al., 2013).

The expression of virulence genes in *S*. *aureus* is regulated under the partial control of the two-component quorum-sensing system encoded by genes at the *agr* locus (Bronner et al., 2004). The P2 transcript (RNAII) encodes the quorum-sensing system, which consists of the following four proteins: AgrB (the secreted protein responsible for the export and processing of AgrD to its active form), AgrD (a signaling peptide), and AgrC-ArgA (a two-component system in which AgrC is the transmembrane receptor histidine kinase and AgrA is the DNA-binding response regulator; Novick et al., 1995; Gilot et al., 2002; Novick, 2003; Gilot and van Leeuwen, 2004). A high cell population density causes the activation of AgrA, which induces the transcription of the P3 promoter. Next, P3 drives the transcription of RNAIII, a regulatory RNA that is both a positive and negative regulator of virulence factor production. The activation of RNAIII transcription in response to an increase in cell population density induces a transition in gene expression correlated with metabolic changes and stress adaptations. Toxin- and extracellular enzyme-encoding genes are positively regulated by the *agr-hld* (δ-lysin gene) system, and the genes coding for protein A (*spa*) and coagulase are negatively regulated (Morfeldt et al., 1988). Protein A has a molecular weight of 42 kDa and is covalently anchored to the peptidoglycan of *S. aureus* (Palmqvist et al., 2002). Ninety percent of the molecule is localized in the cell wall, and 10% is in the bacterial cytoplasm. Protein A is an important virulence factor of *S. aureus* based on its ability to bind to a variety of ligands, including the Fc region of IgG, the von Willebrand factor, tumor necrosis factor receptor-1 (TNFR-1), the Fab-heavy chains of the Vh3 subclass, and the epidermal growth factor receptor (EGFR; Cedergren et al., 1993; Viau and Zouali, 2005; Gómez et al., 2006; O’Seaghdha et al., 2006). An increase in protein A during the post-exponential phase has been observed in *agr*-deleted *S. aureus* strains (Novick, 2003).

In addition, AgrA activation leads to increased transcription of the δ-lysin gene (*hld*), which is located immediately upstream of the *agr* operon (Janzon and Arvidson, 1990). δ-lysin is a small polypeptide of only 26 amino acids. It is secreted without a signal peptide, and it makes cation-selective channels in the phospholipid bilayers (Lee and Birkbeck, 1984). δ-lysin is a virulence factor with lytic activity in a wide range of cells, such as neutrophils, macrophages, mammalian erythrocytes, and bacterial protoplasts, as well as in cellular organelles (Julander et al., 1983).

The aim of this study was to assess *agr* system expression by quantifying *hld* and *spa* expression in multidrug-resistant (MDR) *S. aureus* clinical isolates cultured from the exponential to post-exponential growth phases in the presence of vancomycin. In addition, the *agr* group I–IV polymorphisms were evaluated as a factor that predisposes the permanence and survival of MDR *S. aureus* clinical isolates during nosocomial or hospital-acquired infections in the Hospital Infantil de México Federico Gómez (HIMFG).

## Materials and Methods

### Bacterial Isolates

Thirty-three *S. aureus* from different infections were obtained from a clinical isolates collection at the Central Laboratory of the HIMFG. The *S. aureus* isolates were collected from January 2006 to June 2007. They were cultured on 5% sheep blood agar plates (Becton Dickinson, East Rutherford, NJ, USA) at 37°C under 5% CO_2_ for 24 h and kept at -70°C in skim milk (Becton Dickinson, East Rutherford, NJ, USA).

### Diagnostic Tests for Identifying *S. aureus*

The bacteria were grown on blood agar, and identification was performed using conventional bacteriological techniques, such as colony morphology examination, catalase assays, coagulase assays, Gram staining (Sigma-Aldrich, St. Louis, MO, USA), mannitol fermentation, and Brain Heart Infusion (BHI) broth growth assays (Becton Dickinson, East Rutherford, NJ, USA) with 15% NaCl (MacFadin, 1996).

### Antimicrobial Susceptibility

The antibiotic susceptibility profiles of the *S. aureus* isolates were determined by the Minimum Inhibitory Concentration (MIC) technique with the microdilution method in Mueller-Hinton broth (MH; Becton Dickinson, East Rutherford, NJ, USA), as recommended by the Clinical and Laboratory Standards Institute (2014). The MIC tests were conducted with vancomycin, ciprofloxacin, erythromycin (MP Biomedicals, Solon, OH, USA), clarithromycin (Grünenthal Gmbh, Aachen, Germany), oxacillin, clindamycin, linezolid (Sigma-Aldrich, St. Louis, MO, USA), meropenem (AstraZeneca Pharmaceuticals LP, Wilmington, DE, USA), trimethoprim, sulfamethoxazole (Roche, Basel, Switzerland), and gentamicin (Schering-Plough Pharmaceuticals, Kenilworth, NJ, USA). To identify methicillin-resistant *S. aureus* clinical isolates, the bacteria were tested for oxacillin resistance by the oxacillin-salt screening method. Oxacillin is a more stable antibiotic than methicillin, although they are chemically identical. *S. aureus* strain ATCC 29213 (American Type Culture Collection, Manassas, VA, USA) was used as a positive control.

### Molecular Genotyping Assays

Pulsed-field gel electrophoresis (PFGE) was performed using a previously described protocol (Pereira et al., 2014). The chromosomal DNA from MDR and sensitive *S. aureus* isolates was digested with the Sma I restriction enzyme (Thermo Fisher Scientific Inc., Life Technologies, Grand Island, NY, USA) and subjected to electrophoresis on 1% agarose gels (Promega; Madison, WI, USA) using the following parameters: 200 V (6 v/cm) at 14°C for 21.5 h, with an initial change of 5 s and a final change of 40 s. The gels were stained with 0.5 μg/mL ethidium bromide solution (Sigma-Aldrich, St. Louis, MO, USA) and visualized using a gel imaging system (ChemiDoc^TM^ MP System, Biorad, Hercules, CA, USA). The DNA fragment patterns generated by PFGE were analyzed with NTSY-pc software (version 2.0, Applied Biostatistics, Inc., Port Jefferson, NY, USA; Ramazanzadeh et al., 2013) using the Sørensen–Dice similarity coefficient and the unweighted pair group method with arithmetic mean (UPGMA) clustering approach (Dice, 1945).

### Multiplex Polymerase Chain Reaction (PCR) Conditions

The *S. aureus* clinical isolates were recovered from frozen stock onto BHI agar plates and incubated at 37°C for 18–24 h. Genomic DNA extraction was performed with a Wizard Genomic DNA Purification Kit (Promega, Madison, WI, USA) from a bacterial culture grown in BHI broth. Briefly, the bacterial culture pellet was mixed with TE buffer (10 mM Tris HCl and 1 mM EDTA, pH 8.0), lysozyme (0.25 mg/mL; Sigma-Aldrich, St. Louis, MO, USA), proteinase K (0.0125 mg/mL; Sigma-Aldrich, St. Louis, MO, USA), and lysostaphin (0.062 mg/mL; Sigma-Aldrich, St. Louis, MO, USA). Multiplex polymerase chain reaction (PCR) assays for detecting *agr* polymorphisms (*agr*I, *agr*II, *agr*III, and *agr*IV; **Table 1**) were prepared according to the protocol for Go Taq Green Master Mix (Promega, Madison, WI, USA). Multiplex PCR reactions were prepared in a final volume of 25 μL as follows: 12.5 μL of Go Taq Green Master Mix 2x (Promega, Madison, WI, USA), 2 μL of bacterial DNA (100 ng/μL), 5 μL of *agr* primers (*agr*I, *agr*II, *agr*III, and *agr*IV) at 10 pg/μL, and 5.5 μL of nuclease-free water. The DNA amplification was performed in a Veriti 96-Well Thermal Cycler-Life Technologies (Applied Biosystems, Foster City, CA, USA) with the following parameters: an initial denaturation at 94°C for 5 min followed by 26 amplification cycles (denaturation at 94°C for 30 s, annealing at 55°C for 30 s, and extension at 72°C for 60 s), ending with a final extension at 72°C for 7 min. An external positive control [DNA extracted from *S. aureus* strains USA300 (*agr*I), 1749 (*agr*II), and ATCC 25923 (*agr*III)] and an external negative control (DNase/RNase-free distilled water) were included with each run. PCR amplicons (10 μL) were loaded into a 1.5% (wt/v) agarose gel (Promega, Madison, WI, USA) using a 100 bp DNA ladder (Promega, Madison, WI, USA), and electrophoresis was performed in 1x TAE buffer at 100 V for 1 h. The bands were visualized using a gel imaging system (ChemiDoc^TM^ MP System, Biorad, Hercules, CA, USA).

**Table 1:** Primers used in *agr* and SCC*mec* typing by multiplex PCR.

Gene	Primer	Sequence (5′–3′)	Amplicon size (bp)	Reference
*agr*I	*agr* I-F *agr* I-R	ATGCACATGGTGCACATGC GTCACAAGTACTATAAGCTGCGAT	441	Gilot et al. (2002)
*agr*II	*agr* II-F *agr* II-R	ATGCACATGGTGCACATGC TATTACTAATTGAAAAGTGGCCATAGC	575	Gilot et al. (2002)
*agr*III	*agr* III-F *agr* III-R	ATGCACATGGTGCACATGC GTAATGTAATAGCTTGTAAAAAGTGGCCATAGC	323	Gilot et al. (2002)
*agr*IV	*agr* IV-F *agr* IV-R	ATGCACATGGTGCACATGC CGATAATGCCGTAATACCCG	659	Gilot et al. (2002)
SCC*mec* I	*mec* I-F *mec* I-R	GCTTTAAAGAGTGTCGTTACAGG GTTCTCTCATAGTATGACGTCC	613	Zhang et al. (2005)
SCC*mec* II	*mec* II-F *mec* II-R	CGTTGAAGATGATGAAGCG CGAAATCAATGGTTAATGGACC	398	Zhang et al. (2005)
SCC*mec* III	*mec* III-F *mec* III-R	CCATATTGTGTACGATGCG CCTTAGTTGTCGTAACAGATCG	280	Zhang et al. (2005)
SCC*mec* IVa	*mec* IVa-F *mec* IVa-R	GCCTTATTCGAAGAAACCG CTACTCTTCTGAAAAGCGTCG	776	Zhang et al. (2005)
SCC*mec* V	*mec* V-F *mec* V-R	GAACATTGTTACTTAAATGAGCG TGAAAGTTGTACCCTTGACACC	325	Zhang et al. (2005)
*mecA*	*mec* 147-F *mec* 147-R	GTGAAGATATACCAAGTGATT ATGCGCTATAGATTGAAAGGAT	147	Zhang et al. (2005)

F, forward; R, reverse.

The SCC*mec* genes (I, II, III, and IVa) were characterized by multiplex PCR according to Cázares-Domínguez et al. (2015).

### RNA Extraction from *S. aureus* Clinical Isolates

*Staphylococcus aureus* isolates that were grown overnight were adjusted to an optical density of 0.05 at 600 nm and incubated in BHI with 1 μg/mL vancomycin. The bacterial cultures were grown for 4 h until reaching the exponential phase (OD_600_ of 0.6–0.8) and for 11 h just in the post-exponential phase (OD_600_ of 1.2–1.4). Aliquots of bacterial cultures were harvested by centrifugation at 10,000 × *g* for 3 min at 4°C. Each pellet was washed in an equal volume of TE buffer (10 mM Tris HCl and 1 mM EDTA, pH 8.0) three times and lysed with the same buffer supplemented with 0.25 mg/mL lysozyme, 0.0125 mg/mL proteinase K, and 0.062 mg/mL lysostaphin. Total bacterial RNA was isolated (TRIzol, Life Technologies, Carlsbad, CA, USA) according to the manufacturer’s directions. After purification, contaminating DNA was removed with RNase-free DNase I (2 U/10 μg of total bacterial RNA at 37°C for 30 min). The RNA was then re-purified with RNeasy Minicolumns (Qiagen Incorporated, Ln Valencia, CA, USA). The amount of recovered RNA was determined spectrophotometrically, and the samples were then stored at -80°C.

### Transcriptional Expression Analyses of the *spa* and *hld* Genes

The relative expression levels of the *spa* and *hld* genes were determined by cDNA-PCR. The purified RNA of all *S. aureus* clinical isolates was employed for reverse transcription (RT)-PCR assays with the GeneAmp RNA PCR Kit (Applied Biosystems, Foster City, CA, USA), using specific primers for the *spa* (encoding protein A) and *hld* (encoding a delta toxin) genes (**Table 2**). A GeneAmp RNA PCR Kit was used with 0.2 μg of total RNA per reaction as a template for PCR amplification. Reactions containing *S. aureus* cells alone, only RNA, or no reverse transcriptase were used as negative controls. Specific primers were used for the amplification of 16S RNA, which was used as an internal control (**Table 2**).

**Table 2:** Primers used in *hld* and *spa* expression analysis by RT-PCR.

Gene	Primer sequence (5′–3′)	Product size (bp)	Reference
*spa*	TATCTGGTGGCGTAACACCTG GATGAAGCCGTTACGTTGTTC	322	Goerke et al. (2000)
*hld*	GAAGGAGTGTTTCAATGG TAAGAAAATACATAGCACTGAG	260	Goerke et al. (2000)
*16S*	TCCGGAATTATTGGGCGTAA CCACTTTCCTCTTCTGCACTCA	121	Goerke et al. (2000)

The expression levels of *spa* and *hld* transcripts from *S. aureus* clinical isolates were quantified by densitometric analysis with Bio-Rad image software (Bio-Rad chemi-doc, Quantity one 4.4.1). The data are expressed as the mean ± standard error of the means. A *p*-value of less than 0.05 was considered significant. All experiments were repeated at least three times, and a representative result is shown for each experiment.

### Quantitative Measurements of Protein A

A qualitative screening test for the production of protein A was conducted by an enzyme linked immunosorbent assay (ELISA). Briefly, 96-well plates containing 200 μL of BHI were inoculated with 10 μL (1.5 × 10^8^ bacteria/mL) of bacterial suspensions and incubated at 37°C in the presence or absence of vancomycin to the exponential phase (4 h) and post-exponential phase (11 h). Cell wall-associated protein A was identified using anti-protein A, followed by mouse anti-IgG antibodies, *o*-phenylenediamine (OPD) compounds, and ELISA as previously described (Ohkaru et al., 1995).

### Quantitative Determination by Biofilm Assays

Biofilm formation was quantitatively analyzed according to the protocol described by Erdem et al. (2008). MDR and sensitive *S. aureus* clinical isolates were grown in BHI broth overnight at 37°C. Then, 96-well plates containing 200 μL of BHI were inoculated with 10 μL (1.5 × 10^8^ bacteria/mL) of bacterial suspensions and grown at 37°C in the presence or absence of vancomycin in the exponential phase (4 h) and post-exponential phase (11 h). The biofilms that developed on the surfaces of the wells were gently washed three times with 1x phosphate-buffered saline (PBS; pH 7.4) and fixed with 2% formaldehyde at 4°C overnight. Wells with fixed biofilms were decanted, washed three times with PBS and stained with 200 μL of 1% crystal violet for 30 min. The excess crystal violet was removed, and the plates were washed twice with water. Crystal violet was subsequently solubilized in 70% methanol, and the absorbance was determined at 620 nm. Assays were performed in triplicate and repeated three consecutive times.

## Results

### The Antimicrobial Susceptibility Testing of *S*. *aureus* Clinical Isolates

Thirty-three *S. aureus* clinical isolates were tested for antimicrobial susceptibility. In total, 60.60% (20/33) of *S. aureus* clinical isolates were MDR, 100% (33/33) were sensitive to four antibiotics (vancomycin, trimethoprim/sulfamethoxazole, gentamicin, and linezolid), and 39.39% (13/33) were sensitive to all antimicrobials (**Table 3**).

**Table 3:** MIC analysis for the MDR and sensitive *S. aureus* clinical isolates.

Clinical isolates	Antibiotics (μg/mL)										
	CEC	CEP	VAN	CLA	CIP	STX	OXA	GEN	ERI	MEM	LZN
488H, 428H, 10H, 714H	≥128	≥128	1	≥128	64	0.5	16	1	≥128	64	2
242H, 143H	≥128	≥128	1	≥128	≥128	2	≥128	0.5	≥128	32	4
246H, 175H	≥128	≥128	1	≥128	≥128	1	≥128	0.5	≥128	16	4
882HR	≥128	≥128	1	≥128	64	1	≥128	1	≥128	32	2
425LCR	≥128	≥128	1	≥128	64	0.5	16	0.5	≥128	64	2
330H	≥128	≥128	1	≥128	≥128	2	≥128	1	≥128	32	4
783H	≥128	≥128	1	≥128	≥128	2	≥128	1	≥128	32	4
902H	≥128	≥128	1	≥128	64	1	≥128	2	≥128	32	4
54H	≥128	≥128	0.5	≥128	64	0.5	16	0.5	≥128	32	2
A-32	≥128	≥128	0.5	≥128	≥128	0.5	≥128	0.5	≥128	16	2
828H	8	≥128	1	≥128	64	0.5	16	0.5	≥128	64	2
931H	8	≥128	1	≥128	64	0.5	16	1	≥128	64	2
260H	0.125	64	0.5	32	0.25	0.5	64	0.5	≥128	8	0.5
622H	0.125	≥128	1	0.06	0.25	1	32	0.5	0.25	4	4
299H	0.060	≥128	1	≥128	32	0,5	8	0.5	≥128	0.12	0.5
679H, 318LCR, 573H, 770H, 633H, 779H, 291H, 336H, 18H, 108H, 780H, 493H, 198H	≤0.25	≤8	≤1	≤0.25	≤2	≤1	≤1	≤1	≤1	≤0.125	≤4

**CVR^∗^**	**≥32**	**≥32**	**≥16**	**≥8**	**≥4**	**≥4**	**≥4**	**≥16**	**≥8**	**≥16**	**≥8**

CEC, cefaclor; CEP, cephalothin; VAN, vancomycin; CLA, clarithromycin; CIP, ciprofloxacin; STX, trimethoprim/sulfametoxazole; OXA, oxacillin; GEN, gentamicin; ERI, erythromycin; MEM, meropenem; LZN, linezolid. The MDR *S. aureus* clinical isolates are marked in gray. ^∗^Cut-off values for resistance to MIC (μg/mL; CVR).

### Molecular Typing Analysis of the MDR and Sensitive *S. aureus* Isolates by PFGE

**FIGURE 1:**
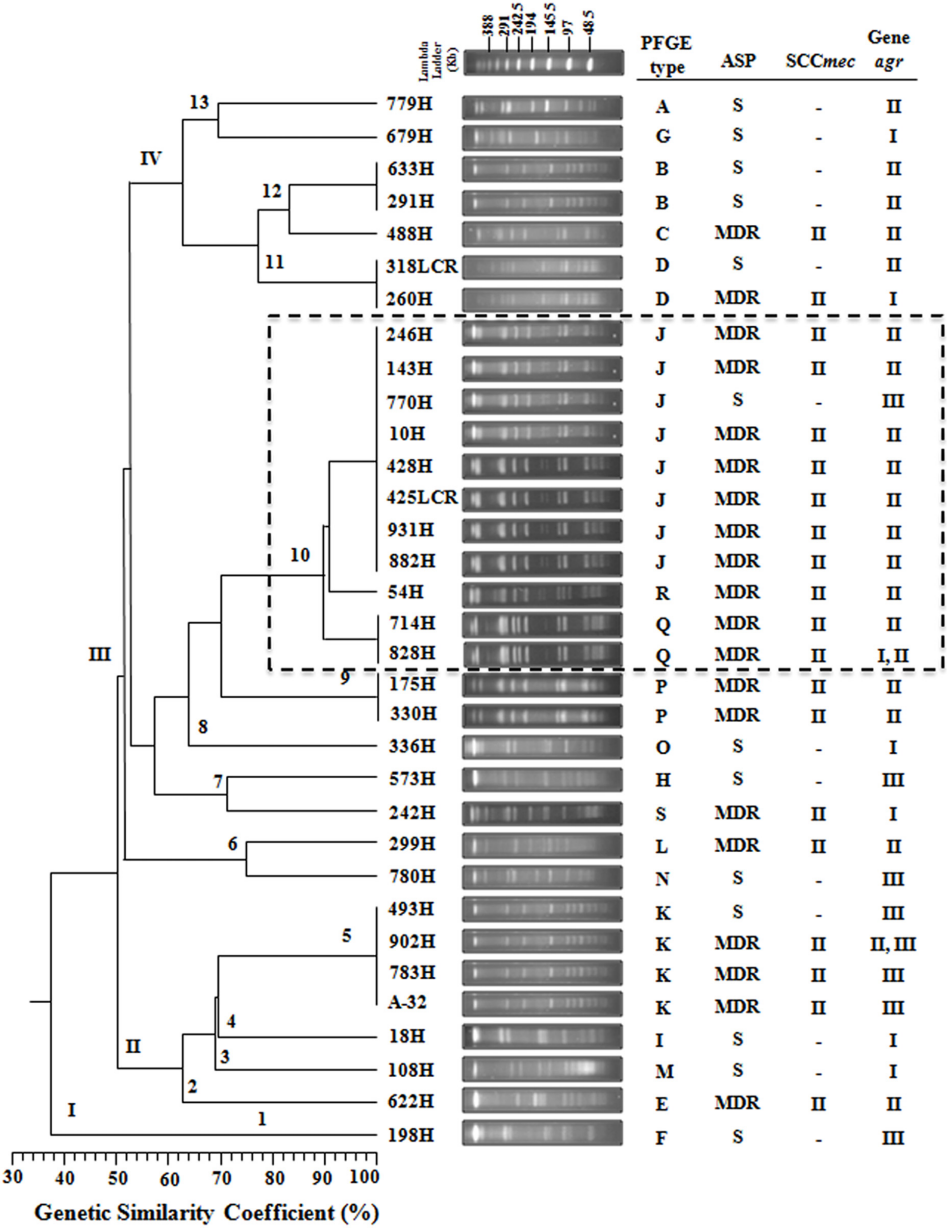
**Dendrogram analysis of PFGE results showing the genetic relationships among the PFGE profiles and the presence of *agr* and SCC*mec* type among the 33 *S. aureus* clinical isolates.** A phylogenetic analysis was conducted using the Sørensen–Dice similarity coefficient in association with the UPGMA algorithm as the grouping method. The dendrogram was evaluated by obtaining the cophenetic correlation coefficient with the Mantel test, which yielded an *r*-value of 0.9077. Antimicrobial susceptibility profile (ASP), multidrug-resistant (MDR), sensitive (S). The dotted line indicates that the clinical isolates that belong to subcluster 10 with >90% genetic similarity.

A total of 19 DNA pulsotypes grouped in four clusters (I–IV) were identified, revealing patterns that consisted of 11–18 DNA fragments ranging in size from 48.5 to 339.5 Kb (**Figure 1**). In total, 3.03% (1/33) of the *S. aureus* clinical isolates were identified as pulsotype F, and cluster I showed 38% genetic similarity when compared with other pulsotypes. Four pulsotypes (E, I, K, and M) belonging to cluster II included 21.21% (7/33) of the *S. aureus* clinical isolates with 64% genetic similarity. In addition, the nine pulsotypes (H, J, L, N, O, P, Q, R, and S) organized in cluster III included 54.55% (18/33) of the *S. aureus* clinical isolates with 56% genetic similarity. In this cluster, the *S. aureus* clinical isolates with >90% genetic similarity were grouped in subcluster 10 and distributed in the following order: two isolates in pulsotype Q, one isolate in pulsotype R, and eight isolates in pulsotype J (**Figure 1**). In addition, 21.21% (7/33) of the *S. aureus* clinical isolates were classified as cluster IV with 62% genetic similarity and distributed as pulsotypes A, B, C, D, and G (**Figure 1**).

Only one *S. aureus* clinical isolate sensitive to all antibiotics was identified (pulsotype F of cluster I; **Figure 1**). Four MDR and three sensitive *S. aureus* clinical isolates were distributed in the four pulsotypes (E, I, K, and M) of cluster II (**Figure 1**). Fourteen MDR and four sensitive *S. aureus* clinical isolates were distributed into the nine pulsotypes (H, J, L, N, O, P, Q, R, and S) of cluster III. Interestingly, seven MDR and one sensitive *S. aureus* clinical isolates that were distributed in pulsotype J were organized in subcluster 10. In addition, two MDR and five sensitive *S. aureus* clinical isolates were distributed over the five pulsotypes (A, B, C, D, and G) of cluster IV (**Figure 1**).

### Distributing *agr*, SCC*mec*, *hld*, and *spa* Genes by Multiplex PCR Endpoint Analysis

**FIGURE 2:**
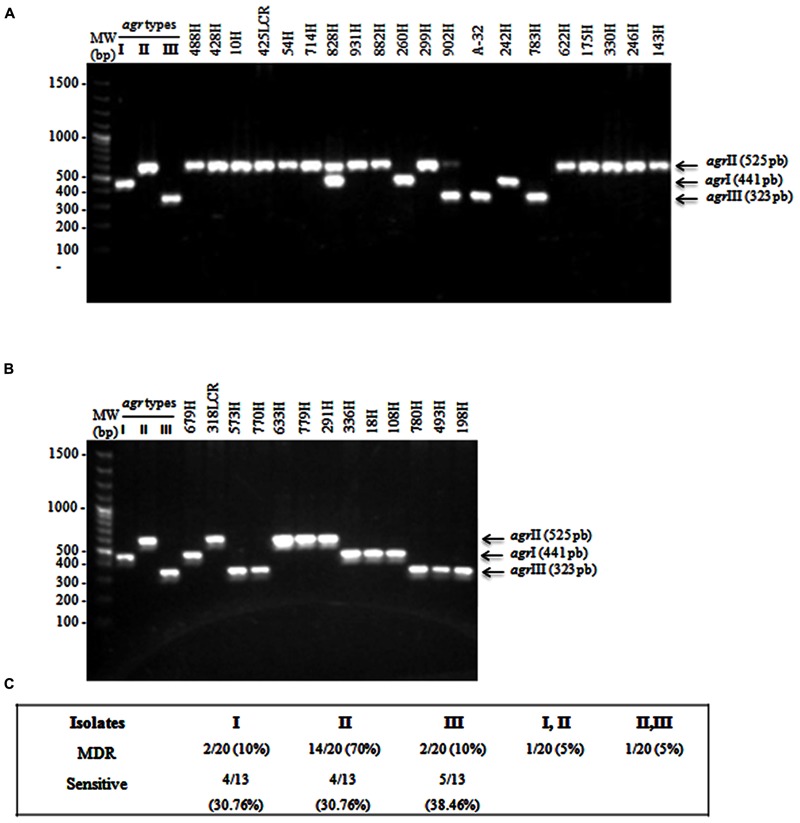
**Multiplex PCR assays for identifying *agr* types. (A)** MDR *S. aureus* clinical isolates showing the *agr*I (441 bp), *agr*II (575 bp), and *agr*III (323 bp) types. **(B)** Sensitive *S. aureus* clinical isolates. **(C)** The percentages of *agr* types that were identified in MDR and sensible *S. aureus* clinical isolates. *S. aureus* strain USA300 (*agr*I), 1749 (*agr*II), and ATCC 25923 (*agr*III) were used as reference strains. MW, molecular weight (bp).

Multiplex PCR amplification assays were performed for *agr*-specific group identification in the selected MDR and sensitive *S. aureus* clinical isolates. Our results showed three different *agr* specificity groups (**Figures 2A,B**), which were identified according to the expected product sizes (**Table 1**). Briefly, the sensitive *S. aureus* clinical isolates were 30.76% (4/13) *agr*I, 30.76% (4/13) *agr*II, and 38.46% (5/13) *agr*III (**Figures 2B,C**). The MDR *S. aureus* clinical isolates were 70% (14/20) *agr*II, 10% (2/20) *agr*I, and 10% (2/20) *agr*III (**Figures 2A,C**). Furthermore, both the *agr*I/II and *agr*II/III polymorphism groups were identified in a single (1/20; 5%) clinical isolate of MDR *S. aureus* (**Figures 2A,C**). The expression of the *agr*IV was not identified in either the MDR or sensitive *S. aureus* clinical isolates.

A 398 bp product corresponding to the SCC*mec* group II polymorphism was present in 60.60% (20/33) of the MDR *S. aureus* clinical isolates that were distributed in the fourth cluster as determined by PFGE. A SCC*mec* II polymorphism was observed in 12.12% (4/33) of the isolates in cluster II, 42.42% (14/33) of the isolates in cluster III, and 6.06% (2/33) of the isolates in cluster IV. However, the SCC*mec* II polymorphism was not identified in cluster I (**Figure 1**). Moreover, SCC*mec* polymorphism types I, III, and IV were not identified in the MDR *S. aureus* clinical isolates (**Figure 1**). It is important to emphasize that the presence of the SCC*mec* gene explains methicillin (oxacillin) resistance; therefore, it was not identified in sensitive *S. aureus* clinical isolates (**Figure 1**).

The frequencies of the *hld* and *spa* genes were determined using specific primers to be 100% (33/33) in MDR and sensitive *S. aureus* clinical isolates. Our results showed two bands, which corresponded to *hld* at 260 bp and *spa* at 322 bp (data not shown).

### *hld* and *spa* Gene Expression as Determined by RT-PCR

**FIGURE 3:**
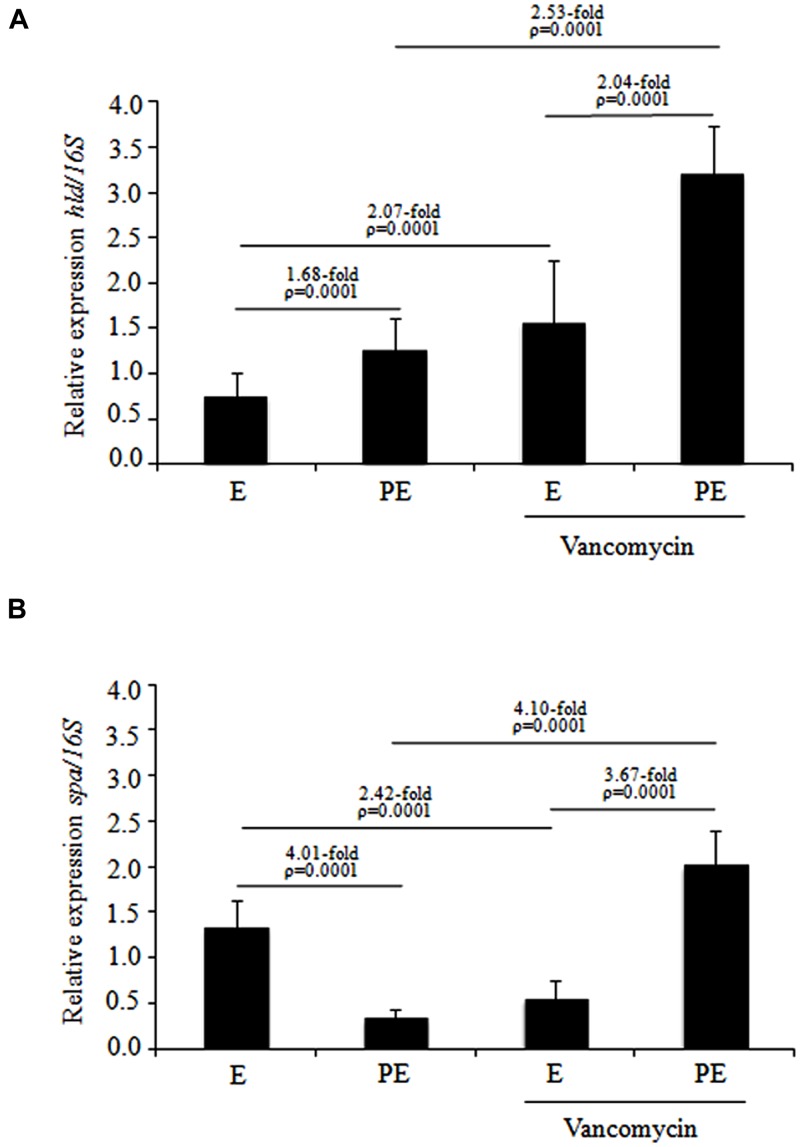
**Expression levels of the *hld* and *spa* genes determined by RT-PCR in MDR *S. aureus* clinical isolates cultured from the exponential to post-exponential growth phases in the presence of vancomycin. (A)** *hld* expression; **(B)** *spa* expression. The densitometric results of the transcripts were quantified using the Quantity One program and analyzed by paired Student’s *t*-tests. E, exponential; PE, post-exponential.

The specific transcripts of the *spa* and *hld* genes produced by the ten MDR *S*. *aureus* isolates that were distributed in subcluster 10 of cluster III were quantified by RT-PCR-densitometry. These MDR *S*. *aureus* isolates were treated with and without vancomycin during two different stages of the growth phase (exponential and post-exponential). The *hld* expression in MDR *S. aureus* clinical isolates showed a significant increase (1.68-fold; *p* = 0.0001) when cultured from the exponential to the post-exponential growth phase in the absence of vancomycin as well as a significant increase (2.04-fold; *p* = 0.0001) when grown in the presence of vancomycin (**Figure 3A**). Interestingly, under the same test conditions, a significant increase (2.07-fold; *p* = 0.0001) in *hld* expression was observed in MDR *S. aureus* clinical isolates in the exponential growth phase treated with vancomycin compared to those treated without vancomycin (**Figure 3A**). In addition, a significant increase (2.53-fold; *p* = 0.0001) in *hld* expression was observed in the MDR *S. aureus* clinical isolates cultured to the post-exponential growth phase when challenged with 1 μg/mL vancomycin compared to without vancomycin challenge (**Figure 3A**). The *hld* expression in sensitive *S. aureus* isolates showed increases from the exponential to the post-exponential growth phase in both the presence and absence of vancomycin (data not shown).

The *spa* expression level showed a significant reduction (4.01-fold; *p* = 0.0001) when the MDR *S. aureus* clinical isolates were cultured from the exponential to post-exponential growth phases in the absence of vancomycin (**Figure 3B**). Interestingly, a remarkable increase (3.67-fold; *p* = 0.0001) in *spa* expression was observed from the exponential to the post-exponential phases in MDR *S. aureus* clinical isolates cultured in the presence of vancomycin (**Figure 3B**). During the exponential phase, a significant reduction in *spa* expression (2.42-fold; *p* = 0.0001) was observed when the MDR *S. aureus* clinical isolates were challenged with vancomycin compared to those grown in the absence of vancomycin; the MDR *S. aureus* clinical isolates in the post-exponential growth phase showed a significant increase (4.10-fold; *p* = 0.0001) in *spa* expression when challenged with vancomycin compared to those grown in the absence of vancomycin (**Figure 3B**). The *spa* expression level did not show a significant changes (reduction and/or increase) when the sensitive *S. aureus* clinical isolates were cultured in the exponential and post-exponential growth phases in the presence or absence of vancomycin (data not shown).

### Protein A Immunodetection by ELISA

**FIGURE 4:**
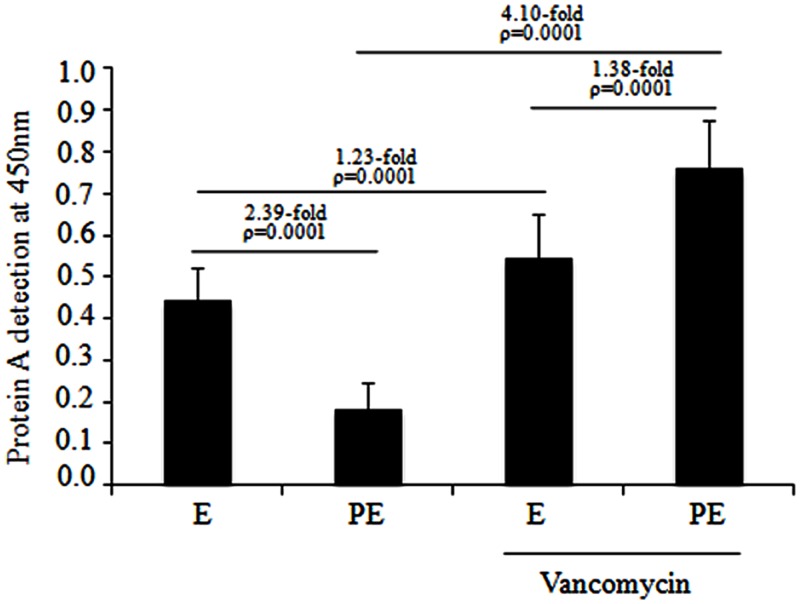
**Protein A production in the MDR *S. aureus* clinical isolates cultured from the exponential to post-exponential growth phases in the presence of vancomycin.** A significant reduction of 2.39-fold (*p* = 0.0001) was observed in the MDR *S. aureus* clinical isolates when they were cultured from the exponential to post-exponential growth phases in the absence of vancomycin and there was a significant increase of 1.38-fold (*p* = 0.0001) when cultured from the exponential to post-exponential growth phase when challenged with 1 μg/mL vancomycin.

Protein A immunodetection assays were performed for the MDR *S. aureus* clinical isolates distributed in subcluster 10 of cluster III. Quantitative analysis showed a significant reduction (2.39-fold; *p* = 0.0001) in protein A expression in the post-exponential phase compared to the exponential phase in the absence of vancomycin (**Figure 4**). Furthermore, a significant increase (1.38-fold; *p* = 0.0001) in protein A expression was observed from the exponential to the post-exponential growth phase in the MDR *S. aureus* clinical isolates cultured the presence of 1 μg/mL vancomycin. Interestingly, a significant increase (4.10-fold; *p* = 0.0001) in protein A expression was observed in these clinical isolates cultured to the post-exponential growth phase in the presence of vancomycin compared to in the absence of vancomycin (**Figure 4**). Likewise, a significant increase (1.23-fold; *p* = 0.0001) in protein A expression was observed in the MDR *S. aureus* clinical isolates that were cultured to the exponential growth phase in the presence of vancomycin compared to in the absence of vancomycin.

### Clinical Strains of MDR and Sensitive *S. aureus* Isolates Produce Biofilms

**FIGURE 5:**
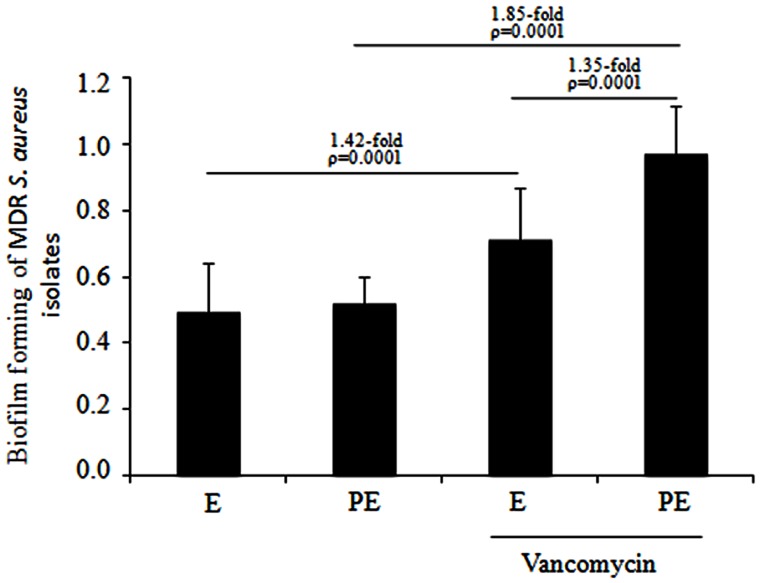
**Biofilm assays of the MDR *S. aureus* clinical isolates when cultured from the exponential to post-exponential growth phases in the presence of vancomycin.** Interestingly, a significant increase (1.35-fold; *p* = 0.0001) in biofilm formation was observed in the MDR *S. aureus* clinical isolates cultured from the exponential to post-exponential growth phases in the presence of the vancomycin.

A crystal-violet biofilm assay was performed for the MDR *S. aureus* clinical isolates that were distributed in subcluster 10 of cluster III. Quantitative analysis showed no difference in biofilm formation when the MDR *S. aureus* clinical isolates were cultured to the exponential and post-exponential growth phases in the absence of vancomycin. In the presence of vancomycin, a significant increase (1.42-fold; *p* = 0.0001) was observed in the biofilm formation of the MDR *S. aureus* clinical isolates cultured to the exponential growth phase and a significant increase (1.85-fold; *p* = 0.0001) was observed in the isolates cultured to the post-exponential growth phase. Furthermore, a significant increase (1.35-fold; *p* = 0.0001) in the biofilm formation of these isolates was observed from the exponential to post-exponential growth phases in the presence of vancomycin (**Figure 5**).

## Discussion

In this study, 100% of *S. aureus* clinical isolates showed sensitive to vancomycin, trimethoprim/sulfamethoxazole, gentamicin, and linezolid. Low rates of resistance to tetracycline, gentamicin, and trimethoprim/sulfamethoxazole in clinical methicillin-resistant staphylococcus aureus (MRSA) isolates have been described in other studies (Bordon et al., 2010; Davis et al., 2013). However, the impressive ability of *S. aureus* to develop resistance against various antibiotics through point mutations, transposons, plasmids, and resistance cassettes is the most likely reason for the rapid increase in antimicrobial resistance worldwide in recent years (Sakoulas and Moellering, 2008). High levels of erythromycin resistance and increasing ciprofloxacin resistance in MRSA clinical isolates have been observed (Davis et al., 2013). The clinical condition, the administration route, and the resistance pattern of the organism are also risk factors that are considered in treating MRSA infections when drugs such as vancomycin, linezolid, daptomycin, clindamycin, and mupirocin are administered (Liu et al., 2011). The increase of vancomycin-resistant *S. aureus* (VRSA) isolates in the United States could be explained by the selective pressure originating from the excessive use of vancomycin to treat MRSA infections (David and Daum, 2010). The high frequency of resistance to the antibiotics tested in our study may reflect the fact that the public health service in Mexico prescribes all of these antibiotics for treating pediatric patients; these drugs are considered to be essential in this health sector.

According to the PFGE analysis, 33.33% (11/33) of the *S. aureus* clinical isolates distributed in subcluster 10 shared 90% similarity. In particular, 50% of the MDR *S. aureus* clinical isolates were distributed in subcluster 10 and showed closely related pulsotypes with three clones that were assigned as J, Q, and R according to the criteria of Tenover et al. (1995). These results suggest that MDR *S. aureus* isolates are associated with patients with infections acquired during their current hospital stay. MRSA isolates with a highly related PFGE type have been associated with an MDR profile to β-lactams, gentamicin, ciprofloxacin, clindamycin, and erythromycin (Velazquez-Meza et al., 2004). In total, 90.90% (10/11) of the *S. aureus* clinical isolates belonging to subcluster 10 were resistant to six antibiotics, namely cephalothin, clarithromycin, ciprofloxacin, oxacillin, erythromycin, and meropenem. Furthermore, 72.72% (8/11) of these clinical isolates were resistant to cefaclor.

Our data showed a high prevalence of the *agr* group II polymorphism, with a PCR-amplified product of 575 kb, in the MDR *S. aureus* clinical isolates belonging to subcluster 10 by PFGE analysis. Several studies performed in Japan and the USA described the *agr* group II polymorphism as the *agr* type that was most frequent in MDR *S. aureus* clinical isolates, and it has been associated with nosocomial infections from pediatric patients (Sakoulas et al., 2003). Similar findings indicated that all *S. aureus* clinical isolates from diverse geographic origins and those recovered from patients undergoing intubation showed the *agr* group II polymorphism (Sakoulas et al., 2002; Goerke et al., 2003; Moise et al., 2004). Furthermore, the *agr* group II polymorphism in MRSA predicts the failure of vancomycin therapy (Moise et al., 2007).

We further evaluated *agr* expression through indirect mechanisms by quantification of *hld* and *spa* expression in MDR *S. aureus* clinical isolates during the exponential and post-exponential growth phases and upon vancomycin challenge. Our RT-PCR analysis showed that *hld* activation occurs during the post-exponential growth phase, resulting in an increase of 1.68-fold without vancomycin and 2.04-fold with vancomycin compared with the exponential phase. The increased *hld* expression in the MDR *S. aureus* clinical isolates of subcluster 10 suggested that the activation of this gene is influenced by the growth phase and vancomycin challenge. Other studies have shown significant increases in *hld* expression at the end of the exponential growth phase by an *agr*-dependent mechanism that is involved in the regulation of virulence genes. *agr*A codes for a protein that can activate *hld* transcription as a response to the growth phase (Janzon and Arvidson, 1990). Our data indicated that the MDR *S. aureus* isolates were stimulated by vancomycin at subinhibitory concentrations, as indicated by increases in *hld* expression. These results indicate that the antibiotic stimulates the *agr* system. In a previous study, the over-expression of the *hld* transcript in vancomycin-resistant *S. aureus* strains associated with activation of the sigma factor was observed when these isolates were exposed to subinhibitory antibiotic concentrations (Chen et al., 2011). Recently, community-associated (CA) MRSA was challenged with subinhibitory concentrations of tetracycline and clindamycin, which had a strong stimulatory effect on the activity of the *agr* operon (Joo et al., 2010). These results, together with the data obtained in our studies, suggest that vancomycin also exerts a strong stimulatory effect on the activity of the *agr* operon. It is important to note that *agr* controls many virulence factors of *S. aureus* and that vancomycin is frequently used in Mexico for treating MRSA infections in pediatric patients.

Interestingly, a reduction in *spa* expression was observed during the exponential to post-exponential growth phases when MDR *S. aureus* clinical isolates were cultured without vancomycin. In contrast, a significant increase (3.67-fold) in *spa* expression was observed when MDR *S. aureus* clinical isolates were only cultured with vancomycin during the post-exponential growth phase. These data suggest that *spa* activation or repression is regulated by the growth phase and that expression of *spa* mRNA is modified by drugs such as vancomycin. In previous studies, CA-MRSA isolates treated with daptomycin and vancomycin showed no change in *spa* mRNA and SpA protein levels (Subrt et al., 2011; Otto et al., 2013). However, in a different study, the expression levels of different virulence factors in the CA-MRSA isolates were suppressed in the presence of clindamycin and linezolid (Otto et al., 2013).

The expression levels of the *spa* gene are directly correlated with protein A production because most of the gene transcript is translated as protein A. The localization of protein A in the cell wall could contribute to biofilm formation in MRSA clinical isolates during the post-exponential phase and when challenged with vancomycin. The XdrA regulator has almost as strong an activating effect on *spa* as SarS, and it acts on the same *spa* operator regions (identified potential *cis*-acting regulatory regions) as SarS or on closely overlapping regions (McCallum et al., 2010). The current evidence suggests that XdrA directly regulates *spa* transcripts independently of other well-characterized regulators. The over-expression of *spa* transcripts and protein A in the MDR *S. aureus* clinical isolates challenged with vancomycin could also be related to the action of the regulator XdrA, which should be examined in future studies. In addition, the variation in the levels of *spa* transcripts during different growth phases is likely due to the influence of other regulators and/or to the characteristics of clinical isolates.

## Conclusion

*Staphylococcus aureus* clinical strains are influenced by intercellular signaling through the *agr* system, which modulates the activation and/or repression of many outer membrane proteins associated with biofilms (Cafiso et al., 2012). Vancomycin is the antibiotic of choice for treating nosocomial infections of MDR *S. aureus* clinical isolates in pediatric patients at the HIMFG. These data provide a direct evidence for evaluating the role of virulence genes (*hld* and *spa*) associated with *agr* regulation and suggest the participation of other regulatory elements; which are include an operon that activates many virulence factors that could be important during infection by this nosocomial pathogen. In our study, the polymorphism *agr*II was associated with nosocomial isolates and was the most prevalent polymorphism in MDR *S. aureus*. Our finding showed that vancomycin modified the *hld* and *spa* expression in the MDR *S. aureus* clinical isolates; suggesting that, vancomycin may regulate alternative systems that jointly participate in the regulation of virulence factors involved in bacteria pathogenesis, which allows spread and adaptation into a hospital environmental.

## Author Contributions

Designed and conceived the experiments: VCD, SAO, ACC, NVG, and JXC. Performed the experiments: VCD, GE, and SAO. Analyzed the data: VCD, SAO, ALO, and GER. Contributed reagents/materials/analysis tools: VCD, SAO, ACC, JJOT, and JXC. Wrote and reviewed the manuscript: VCD, SAO, NVG, and JXC.

## Conflict of Interest Statement

The authors declare that the research was conducted in the absence of any commercial or financial relationships that could be construed as a potential conflict of interest.
